# Spine Stereotactic Body Radiotherapy Outcomes in Patients with Concurrent Brain Metastases

**DOI:** 10.7759/cureus.679

**Published:** 2016-07-11

**Authors:** Rovel J Colaco, Henry S Park, Maxwell S Laurans, Veronica S Chiang, James B Yu, Zain A Husain

**Affiliations:** 1 Therapeutic Radiology, Yale University; 2 Neurosurgery, Yale University; 3 Radiation Oncology, Yale University

**Keywords:** radiosurgery, spine, brain metastases, survival, Stereotactic Radiosurgery

## Abstract

Objectives: Stereotactic body radiotherapy (SBRT) is an emerging technique for maximizing tumor and pain control in selected patients with spinal metastases. Outcomes for those with concurrent brain metastases (CBM) have not been well-described previously. The goal of this study was to compare outcomes for patients with or without CBM treated with spine SBRT.

Methods: Records of all patients treated with SBRT for spine metastases at our institution from January 2008 to January 2014 were reviewed. Chi-square analyses and the Mann-Whitney test were used to assess the association of CBM (defined as brain metastasis present prior to or at the time of spinal SBRT) with potential covariates. The log-rank test and Cox proportional hazards regression were used to evaluate the impact of CBM on overall survival and local control from the time of the first course of spine SBRT.

Results: Seventy-eight patients and a total of 86 SBRT lesions were treated. Median patient age was 60 years (range: 38-84 years); 28.2% had radioresistant histologies. A single fraction was used in 91.0% of treatments. One-year local control was 89.4%, and one-year overall survival was 45.8%. A total of 19 patients (24.4%) had CBM. Among these CBM patients, 18 (94.7%) underwent intracranial radiosurgery and nine (47.4%) were diagnosed synchronously with their spine metastases. Local control was not significantly different between patients with or without CBM on univariable (median: 58 months vs. not reached, p = 0.53) or multivariable analyses (HR 0.52, 95% CI 0.06-4.33). Overall survival was also not significantly different between patients with or without CBM on univariable (median: 7 vs. 11 months, log-rank p = 0.12) or multivariable analyses (HR 1.62, 95% CI 0.87-3.03).

Conclusions: Patients with CBM do not appear to have a statistically significant detriment in clinical outcomes, suggesting that CBM should not necessarily be considered a contraindication for spine SBRT. Although our study is limited by significant heterogeneity in tumor type within our series, future work should focus on the development of reliable survival prognosticators for patients undergoing spinal radiosurgery. Nearly half of the patients with CBM were diagnosed synchronously with their spine metastases, emphasizing the usefulness of obtaining a brain MRI for complete staging prior to spine SBRT.

## Introduction

Stereotactic body radiotherapy (SBRT) is an emerging technique for maximizing tumor and pain control in selected patients with spine metastases [1]. Multiple retrospective and early-phase studies have demonstrated the efficacy and safety of spine SBRT both in patients with no history of previous spinal radiation and in the re-irradiation setting [1-4]. A randomized Phase III study, RTOG 0631, focusing on comparing pain control rates with conventional irradiation and SBRT is currently ongoing [5].  

Principal inclusion criteria for RTOG 0631 and other spinal SBRT studies vary but typically include 1) a solitary spine metastasis, 2) two contiguous spine levels involved, or 3) a maximum of three separate sites where each of the separate sites may have a maximal involvement of two contiguous vertebral bodies. In cases of epidural compression, there is typically at least a 3 mm gap between the spinal cord and the edge of the epidural lesion. Although brain metastases (BM) are not typically considered as exclusion criteria for these trials, principally for reasons outlined in further detail below, in practice, very few, if any, patients with BM are typically included in these studies. 

Outcomes for patients manifesting either spinal or brain metastases have historically been very poor, with median survival times often reported as less than four months both for brain metastases following whole brain radiotherapy (WBRT) [6-7] and for conventional fractionated external beam radiotherapy (EBRT) for spinal metastases [8]. With continuing improvements in systemic therapies, however, patients with spinal and other visceral metastatic disease may now survive for many months or even years [1-4]. Similar outcomes are also now being reported in patients with intracranial metastatic disease, with a median survival approaching 15 months in some series for favorable prognosis BM treated with stereotactic radiosurgery (SRS) [9]. Furthermore, the incidence of BM continues to rise as survival from metastatic cancer improves while up to 40% of patients may develop brain metastases during the course of their disease [10].

Survival from BM has historically been estimated using the recursive partitioning analysis (RPA) classification [11]. Eighty-five percent of patients with BM fall into RPA Category 2, whereas those with vertebral and brain metastases are likely to fall into RPA Class 2 or 3 (estimated survival of four and two months, respectively). The American Society for Radiation Oncology (ASTRO) guidelines recommend that patients considered for spinal SBRT should have an estimated survival greater than three months [12] and have, therefore, traditionally precluded the inclusion of patients with BM in many reported spinal SBRT series. Outcomes for patients with spine metastases and concurrent brain metastases (CBM) have, therefore, not been well described previously. 

A recent update of the RPA to a newly modified graded prognostic assessment (GPA) classification allows more disease-specific prognostication for patients with BM; favorable-prognosis patients with BM from breast cancer can have a median survival of 25 months [11].

In the setting of contemporary advances in therapy and prognostic tools, we sought to compare outcomes for patients with or without CBM treated with spine SBRT at our institution. 

## Materials and methods

In this Institutional Review Board-approved study (Yale University Institutional Review Board, approval number 1112009433), we reviewed records of all patients treated with SBRT for spinal metastasis at our institution from January 2008 to January 2014, with follow-up through to March 2016. Informed patient consent was obtained from all patients.

In addition to the presence of CBM, potential covariates for each patient included age, sex, tumor histology, primary vertebral level (defined as whether the majority of vertebral bodies treated were situated in the cervical vs. thoracic vs. lumbar regions), number of segments treated in the same course, biologically effective dose (BED) assuming α/β = 10 (BED_10_), and whether the spinal metastasis were present at the time of cancer diagnosis. BED_10_ was calculated using the linear-quadratic formula, accounting for the total dose and number of fractions. 

Inclusion criteria for spinal SBRT at our institution were broadly similar to those in the RTOG 0631 study [5] and followed the recommended ASTRO guidelines [12].  Melanoma, renal cell carcinoma, and sarcoma were defined as “radio-resistant” histologies. Patients were routinely staged with computer tomography (CT) scans of the thorax, abdomen, and pelvis prior to treatment, although an MRI (magnetic resonance imaging) scan of the brain was not mandatory for staging prior to spinal SBRT.

Diagnosis of spine metastases within one month of the diagnosis of brain metastases was considered “synchronous” [13]. Information regarding brain metastasis treatment delivered (i.e., Gamma Knife (GK) stereotactic radiosurgery, WBRT, and/or systemic therapy) and any previous treatment with EBRT, surgical resection, kyphoplasty, steroids, immunotherapy, or chemotherapy was collected. No patient received cranial surgery prior to spinal SRS.

Patients were immobilized supine in a stereotactic Elekta BodyFix (Elekta Medical Systems, Stockholm, Sweden), body frame for lesions T5 and below and a Brainlab mask (Novalis Radiosurgery, Munich, Germany) for patients with lesions at T4 and above. CT myelograms were used for simulation for patients with lesions located above the termination of the spinal cord. These were performed with the patient in treatment position with 1.25 mm slice thickness with imaging of the entire spine.

For lesions cauda level or below, a standard non-contrast CT was used for simulation. MRI scan with contrast incorporating at least two spinal segments above and below the treatment volume was performed and fused with the CT simulation scan for the purposes of target volume delineation. Images were then transferred to the Brainlab (Novalis, Inc.) system for planning purposes. Critical structures, including the spinal cord, thecal sac, and/or cauda equina, were contoured. For patients undergoing single-fraction radiosurgery with no previous history of previous spinal radiotherapy, the spinal cord dose was constrained to a maximum dose to the cord of 14 Gy with a thecal sac volume receiving 10 Gy (V10) of < 10%. For patients with a history of previous spinal radiotherapy, tolerance doses were based on the published guidelines by Sahgal, et al. [14].

Gross tumor volume (GTV) was defined as gross visible tumor on the planning CT and MRI scans. A modified clinical target volume (CTV) was created based on the International Spine Radiosurgery Consortium Consensus Guidelines for Target Volume Definition in Spinal Stereotactic Radiosurgery [15]. An additional 1 mm expansion (with zero margins posteriorly) was added to form the planning target volume (PTV). A nine-field step-and-shoot coplanar IMRT plan was created using the Brainlab planning system. Patients had an initial clinical follow-up at eight to 10 weeks post-spinal SBRT and subsequently had an MRI and clinical follow-up every three months starting from 12 weeks post-spinal SBRT. Local control was defined as the absence of radiological changes suspicious of recurrence of disease on follow-up imaging. 

Overall survival and local control were calculated from the time of the first course of spine SBRT. Chi-square analyses and the Mann-Whitney rank-sum test were used to assess the association of CBM with the demographic and clinicopathologic covariates. Age was dichotomized at the median. Kaplan-Meier analysis, the log-rank test, and multivariable Cox proportional hazards modeling were used to evaluate the impact of the CBM on the time-dependent outcomes of overall survival and local control. Spinal surgery performed at the irradiated tumor site at any time prior to a patient receiving spinal SBRT was included as a separate variable in the analysis. If a patient received more than one course of SBRT, the patient was included only once in the overall survival analyses (time from the first course to death or last follow-up) in order to prevent artificially inflating our survival data by counting the same patient multiple times. All SBRT courses were included in local control analyses.

Factors associated with a p-value < 0.10 in univariable analyses were included in multivariable analyses using the backward conditional stepwise approach. A two-sided p-value < 0.05 was used to determine statistical significance. All analyses were performed using STATA SE version 13.1 (College Station, TX).

## Results

We included 78 patients who underwent a total of 86 courses of spine SBRT. Complete patient characteristics are detailed in Table 1.

**Table 1:** Patient Characteristics Overall demographic data and patient characteristics (n = 78). BED10: biologically effective dose assuming α/β=10

Characteristic	Number of Patients (%)
Age, median (range)	60 years (38-84 years)
Sex	
Male	32 (41.0)
Female	46 (59.0)
Primary histology	
Non-small cell lung carcinoma	25 (32.1)
Breast carcinoma	15 (19.2)
Melanoma	9 (11.5)
Renal cell carcinoma	13 (16.7)
Sarcoma	11 (14.1)
Other	5 (6.4)
Primary vertebral level	
Cervical	10 (12.8)
Thoracic	42 (53.9)
Lumbar / Sacral	26 (33.3)
Multiple segments treated	
Yes	26 (33.3)
No	52 (66.7)
BED_10_, Gy (median, range)	50.4 Gy (20.0-81.6 Gy)
Total dose, Gy	
10	1 (1.3)
12	1 (1.3)
14	4 (5.1)
16	10 (12.8)
18	26 (33.3)
20	14 (18.0)
22	6 (7.7)
23	2 (2.6)
24	9 (11.5)
27	5 (6.4)
Number of fractions	
1	71 (91.0)
2	1 (1.3)
3	6 (7.7)
Immunotherapy/targeted therapy	
Yes	25 (32.0)
No	53 (68.0)
Metastatic to spine at time of cancer diagnosis	
Yes	33 (42.3)
No	43 (55.1)
Unknown	2 (2.6)
Concurrent brain metastases	
Yes	19 (24.4)
No	59 (75.6)

Median patient age was 60 years (range: 38 to 84 years), 59.0% were female, and 28.2% had radio-resistant histologies. A single fraction was used in 91.0% of treatments. The median BED_10 _was 50.4 Gy (18 Gy in 1 fraction), ranging from 20.0 Gy (10 Gy in 1 fraction) to 81.6 Gy (24 Gy in 1 fraction). 

Median follow-up in this study was 30 months for alive patients, with a one-year overall survival of 45.8%, a two-year overall survival of 31.6%, and median overall survival of nine months. Median follow-up for local control was six months with one-year local control of 89.4% and two-year local control of 80.3%. 

Nineteen out of 78 patients (24.4%) were also diagnosed with CBM. Brain metastases were single in three patients and multiple (range: 2 - 8) in 16 patients. Brain metastases were diagnosed synchronously with spine metastases in nine patients and were diagnosed and treated prior to spine metastases in 10 patients. Eighteen received GK without upfront WBRT and one received immunotherapy alone without upfront radiotherapy. Compared to those without CBM, patients with CBM were more likely to have radio-resistant histology (45.5% vs. 16.1%, p = 0.007) and to receive immunotherapy/targeted therapy (48.0% vs. 16.2%, p = 0.001). All other characteristics were similar between the two groups (Table 2).

**Table 2:** Concurrent Brain Metastases (CBM) Vs. No Concurrent Brain Metastases Patient characteristics and demographic data for patients with and without concurrent brain metastases (CBM) (n = 78). BED10: biologically effective dose assuming α/β=10

*C*haracteristic	CBM (n, %) (n = 19)	No CBM (n, %) (n = 59)	P-value
Age, median (range)	55 years (44-83 years)	61 years (38-84 years)	0.054
Age			0.148
< 60 years	12 (31.6)	26 (68.4)	
≥ 60 years	7 (17.5)	33 (82.5)	
Sex			0.134
Male	5 (15.6)	27 (84.4)	
Female	14 (30.4)	32 (69.6)	
Primary histology			0.007
Radioresistant	10 (45.5)	12 (54.5)	
Non-radioresistant	9 (16.1)	47 (83.9)	
Primary vertebral level (Reference: Thoracic)			0.684
Cervical	10 (100.0)	0 (0.0)	
Thoracic	34 (73.9)	12 (26.1)	
Lumbar/Sacral	22 (73.3)	8 (26.7)	
Multiple segments treated			0.192
Yes	15 (28.9)	37 (71.1)	
No	4 (15.4)	22 (84.6)	
BED_10_, median	50.4 Gy	50.4 Gy	0.995
BED_10_			0.684
≤50.4 Gy	11 (26.2)	31 (73.8)	
>50.4 Gy	8 (22.2)	28 (77.8)	
Immunotherapy/targeted therapy			0.001
Yes	12 (48.0)	13 (52.0)	
No	7 (13.2)	46 (86.8)	
Metastatic to spine at time of cancer diagnosis			0.295
Yes	10 (30.3)	23 (69.7)	
No or Unknown	9 (20.0)	36 (80.0)	

**Figure 1:**
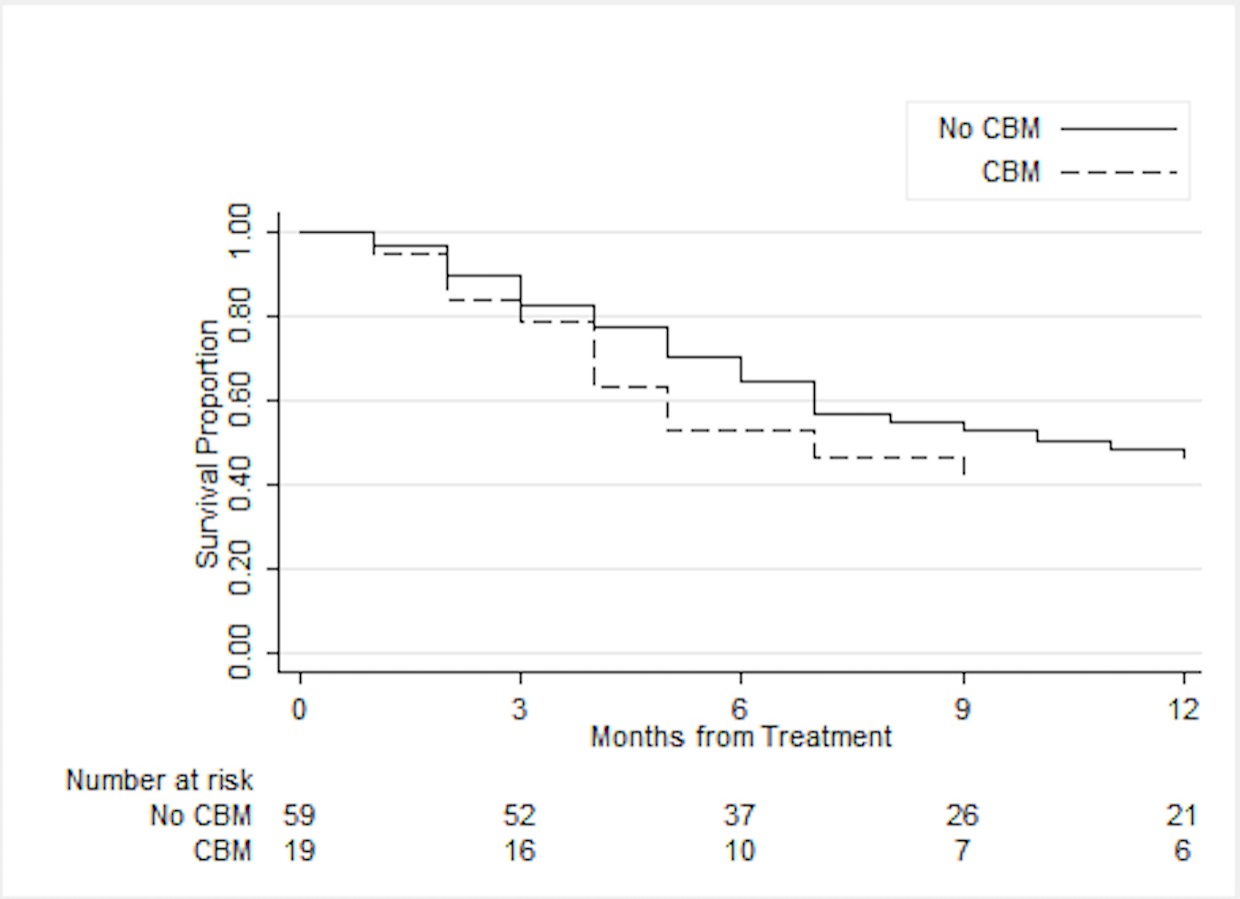
Overall Survival Overall survival for patients with or without concurrent brain metastases (CBM) (n = 78).

Patients with or without CBM achieved similar overall survival on univariable analysis (40.1% vs. 47.7% at one year, log-rank p = 0.12) (Figure 1). 

Patients with radio-resistant histology were more likely to have CBM than those with non-radio-resistant histology (45.5% vs. 16.1%, p = 0.007). Similarly, patients receiving immunotherapy or targeted therapy were more likely to have CBM than those who did not receive those medications previously (48.0% vs. 13.2%, p = 0.001). Since radio-resistant histology and immunotherapy/targeted therapy were highly collinear with each other (p < 0.001), only radio-resistant histology was included in the multivariable analyses. Cox proportional hazards regression continued to show no significant survival difference between patients with or without CBM (HR 1.62, 95% CI 0.87-3.03, p = 0.13). 

**Figure 2:**
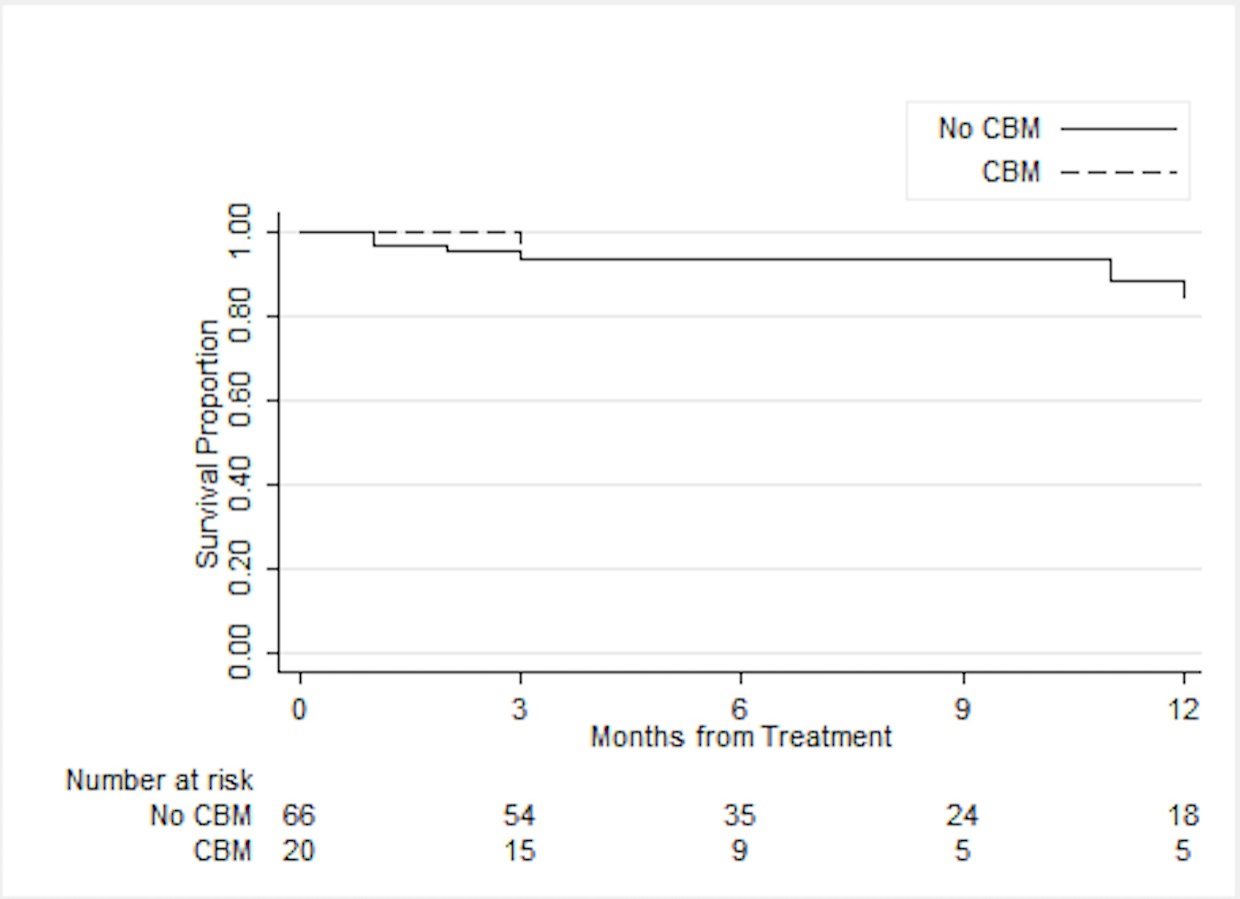
Local Control Local control for patients with or without concurrent brain metastases (CBM) (n = 86).

Similarly, patients with or without CBM achieved similar local control on univariable analysis (92.0% vs. 88.7% at one year, log-rank p = 0.53) and on multivariable analysis (HR 0.52, 95% CI 0.06-4.33, p = 0.55) (Figure 2). 

Eleven patients (14.1%) had undergone previous spinal surgery prior to SBRT. Previous spinal EBRT was delivered to 21 patients (26.9%). There was no significant difference in CBM incidence among those with vs. without prior EBRT (19.0% vs. 26.3%, p = 0.569). There was also no significant difference in survival among patients with vs. without prior EBRT (median: 7 vs. 9 months, log-rank p = 0.671).

## Discussion

SBRT for spinal metastases has rapidly gained popularity over the course of the past decade. Our institution’s early experience has demonstrated excellent overall survival and local control outcomes, which compares favorably to other previously reported large-scale case series in the literature, with a one-year lesional control rate of 90% [5, 16-18]. 

A salient finding in the series of patients reported here was that patients with CBM did not suffer significantly inferior survival compared to those without CBM. While a significant body of evidence demonstrating the efficacy of spine SBRT already exists, our findings fill a knowledge gap on the optimal selection of SBRT patients. Historically, given the poor outcomes faced by patients with BM, many practitioners have been uncertain as to the usefulness of spine SBRT (a significantly more labor- and cost-intensive process for the medical system than conventionally fractionated external beam radiotherapy) in this setting. Furthermore, there is a paucity of reported outcomes in the literature for patients with concurrent brain and spinal metastases. The poor prognosis from BM, along with the fact that many patients with BM were historically likely to present with poor performance status, may be responsible for this fact.

Further development and refinement of predictive tools are needed to identify patients with BM and spinal metastases who will obtain the greatest benefit from spinal SBRT. In this regard, there may be a benefit in establishing a scoring system similar to the aforementioned GPA classification for BM or the Spinal Instability Score (SINS) for predicting the risk of spinal instability in patients with spinal metastatic disease. The Cleveland Clinic recently performed an RPA for patients undergoing SBRT at their institution for spinal metastases [19] to develop a prognostic index specifically for patients undergoing spinal SBRT. Patients were split into three RPA classes depending on the Karnofsky performance status (KPS), time from primary diagnosis (TPD) to presentation for spinal SBRT, and age < 70. In this analysis, 51.1% of patients had extraosseous metastatic disease, although it was not stated what proportion, if any, of these patients had BM. However, patients in the RPA Class 1 (KPS > 70 and TPD > 30 months) had a median survival of 21.1 months. There have been other reports of patients surviving at least this length of time with BM [20], reinforcing the concept that the presence of BM should not necessarily preclude spinal SBRT in this group of patients.

The finding of similar rates of one-year survival between BM and non-BM patients is predicated on an aggressive approach to intracranial metastatic disease. At our institution, brain metastases are typically treated aggressively with SRS upfront, with active follow-up with an MRI scan with and without contrast every six to 12 weeks. Whole brain radiotherapy is typically reserved as salvage treatment, an approach that is supported by multiple phase III clinical trials [21-25].

With the development and increasing availability of more sophisticated and precise methods of radiation delivery for SRS and SBRT, coupled with improvements in treatment outcomes for visceral metastatic disease in a number of cancer sites [26-29], the findings of this study that overall survival was not significantly inferior in patients with CBM undergoing spinal SBRT would seem to suggest that, in appropriately selected cases, CBM should not necessarily preclude patients from being considered for spinal SBRT. Furthermore, nearly half of the patients in our series with CBM were diagnosed synchronously with their spine metastases. Therefore, we would also recommend consideration of concurrent MR imaging of the brain in all patients being evaluated for spine SBRT.

Limitations of this study include those inherent to a small single-institution retrospective analysis. It is possible that our study may be underpowered to detect a true difference in overall survival and local control among patients with or without CBM. In addition, it is difficult to ascertain whether or not our findings are generalizable to patients treated at other institutions that take a less aggressive approach to the management of brain metastases or to the selection of patients eligible for spine SBRT.

## Conclusions

In conclusion, we have found that patients with CBM treated with spine SBRT do not appear to have a statistically significant inferior overall survival when the brain metastases are also treated aggressively. Our data suggests that CBM should not be considered a contraindication for spine SBRT. Although our study is limited by significant tumor heterogeneity within our small sample, future work should focus on the development of prognostic indices that could better predict survival for patients being considered for spine SBRT.
